# Modeling the Role of Weather and Pilgrimage Variables on Dengue Fever Incidence in Saudi Arabia

**DOI:** 10.3390/pathogens13030214

**Published:** 2024-02-28

**Authors:** Kholood K. Altassan, Cory W. Morin, Jeremy J. Hess

**Affiliations:** 1 Department of Family and Community Medicine, King Saud University, Riyadh 11421, Saudi Arabia; 2 Department of Environmental and Occupational Health, University of Washington, Seattle, WA 98195, USA; cwmorin@uw.edu; 3 Department of Emergency Medicine, University of Washington, Seattle, WA 98195, USA; jjhess@uw.edu

**Keywords:** dengue fever, Saudi Arabia, vector-borne disease, predictive models, machine learning

## Abstract

The first case of dengue fever (DF) in Saudi Arabia appeared in 1993 but by 2022, DF incidence was 11 per 100,000 people. Climatologic and population factors, such as the annual Hajj, likely contribute to DF’s epidemiology in Saudi Arabia. In this study, we assess the impact of these variables on the DF burden of disease in Saudi Arabia and we attempt to create robust DF predictive models. Using 10 years of DF, weather, and pilgrimage data, we conducted a bivariate analysis investigating the role of weather and pilgrimage variables on DF incidence. We also compared the abilities of three different predictive models. Amongst weather variables, temperature and humidity had the strongest associations with DF incidence, while rainfall showed little to no significant relationship. Pilgrimage variables did not have strong associations with DF incidence. The random forest model had the highest predictive ability (R^2^ = 0.62) when previous DF data were withheld, and the ARIMA model was the best (R^2^ = 0.78) when previous DF data were incorporated. We found that a nonlinear machine-learning model incorporating temperature and humidity variables had the best prediction accuracy for DF, regardless of the availability of previous DF data. This finding can inform DF early warning systems and preparedness in Saudi Arabia.

## 1. Introduction

Dengue fever (DF) is a potentially life-threatening viral disease transmitted by *Aedes* spp. mosquitoes with an estimated disease burden of over 100 million infections a year [1,2]. The mosquito vectors breed in small bodies of stagnant water, particularly in water storage containers around homes. More recently, the range of the disease has expanded geographically [3], resulting in increasing risk of disease in 129 countries. Early detection and management are key to preventing mortality [1].

Saudi Arabia has one of the largest DF burdens in the Middle East. The first documented case appeared in Jeddah in late 1993 [4]. By March 1994, the Disease Control Division had initiated a dengue surveillance system that recorded 289 cases that year [5]. Sporadic outbreaks occurred in ensuing years, each with no more than 15 cases annually [6]. However, between 2004 and 2015, significantly larger outbreaks occurred, primarily during the rainy season, extending beyond Jeddah into the nearby cities of Makkah, Al-Madinah, Jizan, and Najran, and led the Saudi Ministry of Health to declare DF endemic in the western region of Saudi Arabia [7]. In 2022, 3647 cases of DF were reported with an incidence rate of 11 per 100,000 person-years [8].

The dramatic increase in DF globally over the past 50 years has been attributed to increased urbanization, migration, erratic water supplies, and geographically expanding vector populations associated with climate change, among other factors [7,9]. DF transmission generally follows a seasonal pattern and is highly sensitive to temperature, rainfall, and humidity [9,10,11,12,13]. Temperature influences both the physiology and behavior of the vectors and viral replication rate [9,14,15]; several statistical models have successfully predicted these relationships [9,16,17,18,19]. DF may also be governed by seasonal precipitation as rainfall provides pockets of stagnant water around dwellings [11]. Although humid conditions generally coincide with rainfall, often ambient humidity is enough to create the necessary conditions for *Aedes aegypti* proliferation, by increasing the longevity of female mosquitoes and preventing the desiccation of mosquito ova. Hales et al., (2002) found that average annual vapor pressure was the strongest predictor of DF distribution [20]. Favorable weather conditions can also help imported cases of DF become local epidemics [21].

Climatologic and population factors likely contribute to DF’s epidemiology in Saudi Arabia. While arid, conditions in some areas have allowed DF to become endemic, with a seasonal pattern peaking in the wetter spring (March–May) and a smaller second peak in November and December [22]. This pattern is likely related to the seasonal abundance of the mosquito vectors [23]. The second peak might be attributed to the lower temperatures that are optimal for DF transmission [23]. Population makeup also plays a role. A third of the country’s approximately 30 million are foreign workers [24]. The country hosts over 8 million visiting Muslim pilgrims annually in Makkah, arriving primarily through Jeddah [25], where the climate was particularly favorable for DENV introduction and emergence and persistence remains high [7]. Inter-regional population movement, particularly during the annual Hajj and Umrah pilgrimages, increases disease importation risk [26]. Between 1.5 and 2.5 million pilgrims from over 180 different countries participate in the week-long Hajj [27], most from countries where DF is endemic [28,29]. Millions of pilgrims also travel to Makkah to perform the Umrah pilgrimage. These mass gathering events further drive DENV serotype mixing and transmission, [22,26,28]. As the Hajj falls on the 12th month of the Islamic lunar calendar, the seasonal variability of the event complicates DF transmission dynamics [27].

Despite rich evidence linking weather and climate with DF globally, linkages in Saudi Arabia remain largely unexplored. Further understanding of interactions between weather and demographic factors is needed to anticipate the possible impacts of climate change on dengue incidence [11]. Additionally, understanding the role of pilgrims in the original and continuous importation of dengue virus would improve health system preparedness [7].The World Health Organization (WHO) has emphasized the importance of identifying the factors, particularly weather variables, that may act as leading indicators of DF outbreaks. Predictive models in other locations highlight the importance of these indicators and the potential of predictive modeling to minimize the burden of DF [30]. Recently, Siddiq et al. attempted to predict the geospatial clustering of DF in Jeddah. They used annual and monthly weather variables and environmental variables but did not incorporate any population factors into their models [31].

The objectives of this study are (1) to examine and quantify the relationships between weather, pilgrimage events, and DF in Saudi Arabia, (2) to determine the best statistical modeling approach for DF prediction there, and (3) to utilize this information to create a predictive model for DF incidence.

Research efforts investigating the factors responsible for DF emergence and spread within Saudi Arabia are limited, possibly due to lack of consistent publicly available datasets. We were able to obtain electronic DF data from three cities in Saudi Arabia for a period of 10 years. To our knowledge, this is the first research effort utilizing this rich resource. This study is also the one of the first attempts to predict DF incidence in the Arabian Peninsula using an empirical model. DF in this region presents a unique context as the area is non-tropical and known for its arid climate. It further poses a novel question pertaining to the effect of hosting the Hajj and Umrah pilgrimages on DF epidemiology.

We evaluate the weather variables that can be used to predict DF in Saudi Arabia based on lagged observations and compare three different modeling approaches previously utilized in other geographic regions.

## 2. Materials and Methods

### 2.1. Data Collection

DF data: We obtained electronic weekly DF records for the geographical areas Jeddah, Makkah, and Jizan. Case reporting of hemorrhagic disease is mandatory. All suspected DF cases were included from 2009 to 2018 for Jeddah, and 2012 to 2018 for Makkah and Jizan.

Weather data: We used the GLDAS Noah Land Surface Model L4 3 Hourly 0.25 × 0.25 degree data subsets provided by Goddard Earth Sciences Data and Information Services Center. Weekly measurements of temperature, rainfall, and relative humidity were produced by aggregating GLDAS 3-hourly measurements. The geographical coordinates selected, as per Google search, were 21.375°, 39.375° for Jeddah, and 21.375°, 39.875° for Makkah. The aggregated temperature variables included the weekly *mean*, *mean minimum*, *mean maximum*, *minimum minimum* (the lowest temperature measured in a week), and *maximum maximum* in degrees Celsius (°C). Similar statistics were generated for relative humidity variables as percentages. The aggregated precipitation variables included *total rainfall* and *average rainfall* in mm/day, and *number of rainy days*.

Pilgrimage data: The annual number of pilgrims was obtained from the Saudi General Authority for Statistics. The annual timing of the Hajj was defined as the Gregorian dates coinciding with the 6th of Thul Hijjah to the 13th. The timing of Ramadan, which is the 9th month of the lunar calendar, was calculated as the 11th–14th weeks preceding the week of the Hajj. Pilgrimage variables included the *number of pilgrims* and *proportion of foreign pilgrims* of the previous year, as well as the *week of Hajj* and *month of Ramadan* as binary variables.

### 2.2. Analysis and Model Selection

We analyzed each geographic area separately using RStudio Data Analytics Software version (1.2.1335). Figures demonstrating correlations between DF cases and weather and pilgrimage variables were generated using Microsoft Excel. Variables were selected based on the Pearson correlation coefficients (*r*) and bivariate analyses for each city investigating their relationships with weekly DF case counts. Similar to the approach taken in other studies [30], each weather variable was tested at 1–8-week lags resulting in a total 104 weather variables tested. Four pilgrimage variables were also analyzed with the *Hajj week* and *month of Ramadan* tested at 1–12-week lags, and one selected from each based on the relationship strength, the intrinsic and extrinsic incubation periods, and the time necessary for imported dengue viruses to circulate in the environment. The variables of the *number of pilgrims* and *proportion of foreign pilgrims* for the previous year were included only if they were found to be significant (*p* ≤ 0.05). Additionally, the *year* and *number of cases the previous week* were assessed for inclusion. Two covariate groups reflecting real-world scenarios and limitations were created for inclusion in the models. Group 1 only included the *year* and the statistically significant weather and pilgrimage variables with the highest *r* measurements in their respective categories. Group 2 included the variables from Group 1 and the variable of the *number of cases the previous week.*

We employed three regression modeling methods: a generalized Poisson linear multivariate regression with variables removed in a backward stepwise approach and model fit determined using the Akaike information criteria (AIC); an auto regressive integrated moving average (ARIMA) regression, adding external regressors from the covariate groups, with a non-seasonal ARIMA determined to be the best fitting model using AIC; and a random forest (RF) regression specified to use 750 trees.

### 2.3. Model Validation and Assessment

Throughout the analysis, we utilized an iterative holdout method, dividing the dataset into training and testing subsets, withholding one year of data to test the model and leading to ten iterations of model training and validation. We evaluated the models’ performance based on the R^2^ and root mean square error (RMSE) calculated based on the predicted and observed numbers of cases, with a higher R^2^ and lower RMSE indicating a better performing model. We also visually inspected the model residuals for patterns.

## 3. Results

**Figure 1:**
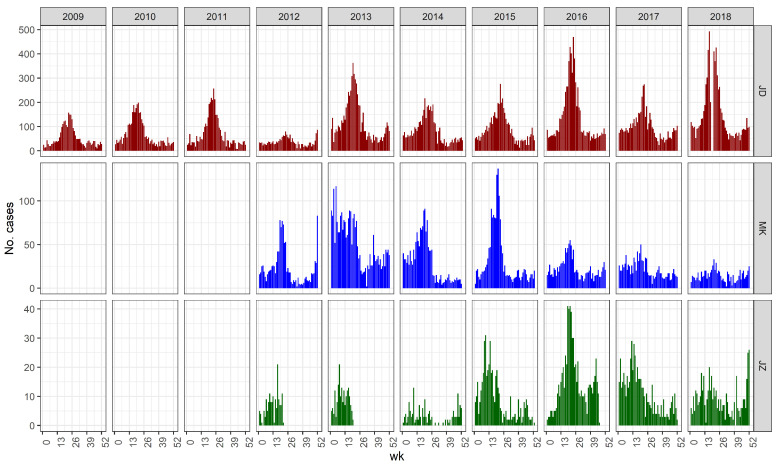
Seasonal distribution of DF cases in Jeddah, Makkah, and Jizan from 2009 to 2018.

Final analyses were performed on both Jeddah and Makkah; Jizan was excluded because of significant periods with missing data (Figure 1). Here, we present the results for Jeddah only. Jeddah is the largest of the three cities, home to the main airport through which pilgrims travel, where the first DF outbreaks began, and has a higher number of cases than the other areas and the longest time-series. The figures describing the results for Makkah can be found in Technical Appendix B.

### 3.1. Descriptive and Trend Analyses

**Figure 2:**
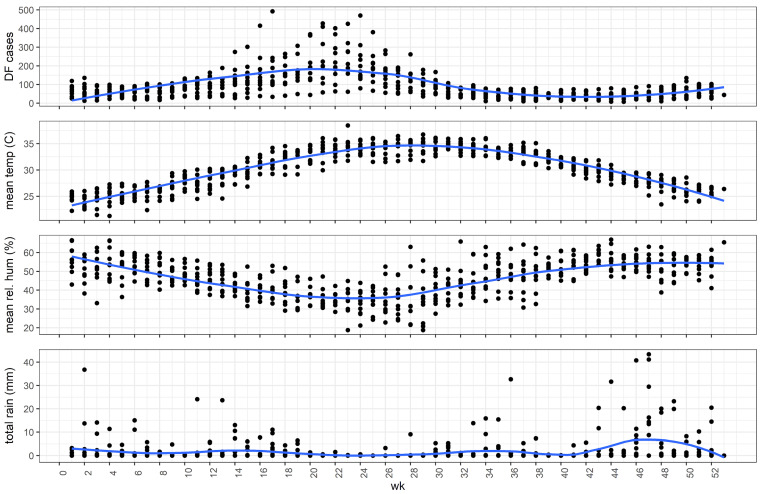
Distribution of DF cases, average temperature, relative humidity, and total rainfall throughout the year in Jeddah based on measurements for 2009 to 2018. The blue line represents the Locally Weighted Scatterplot Smoothing (LOWESS).

**Figure 3:**
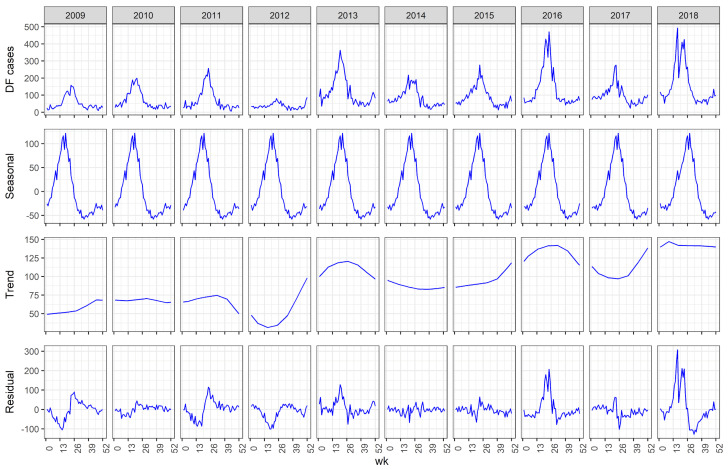
Distribution of DF cases in Jeddah decomposed into a seasonal pattern from 2009 to 2018.

DF incidence showed a seasonal pattern with a large peak in the late spring (Weeks 22 to 30) and another smaller peak in the early winter peak (Weeks 49 to 53; Figure 1). Jeddah’s average temperatures, precipitation, and humidity patterns are shown in Figure 2. Annual DF incidence has shown an overall upward trend since electronic reporting began in 2009 with small dips in the curve every couple of years (Figure 3).

### 3.2. Bivariate Analyses between DF and Independent Variables

#### 3.2.1. Correlations between DF Case Counts and Weather Variables

**Figure 4:**
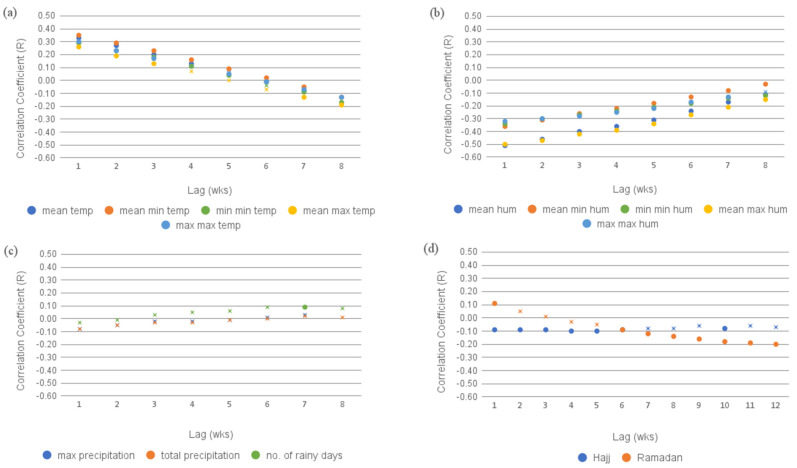
Results of the univariate analysis between DF cases and weather and population variables in the city of Jeddah between 2009 and 2018. (**a**) Correlation with temperature variables. (**b**) Correlation with humidity variables. (**c**) Correlation with precipitation variables. (**d**) Correlation with pilgrimage variables. Non-significant correlations (*p*-value = 0.05) are represented with an “X”.

Temperature: We observed a positive correlation that decreased with a lag time of 5–6 weeks and then became negative. The strongest correlation overall was a moderately strong significant positive association between the *average minimum weekly temperature* and cases at a 1-week lag (r = 0.35). The strongest negative correlation was between the *average maximum temperature* at an 8-week lag (r = −0.19) (Figure 4a).

Humidity: The strength of the observed negative correlation decreased with a lag time up to 8 weeks. The strongest overall correlation was a moderately strong significant negative association between the *average relative humidity* and cases at a 1-week lag (r = −0.51) (Figure 4b).

Precipitation: The *number of rainy days* at a 7-week lag showed a positive significant association (r = 0.09) (Figure 4c).

#### 3.2.2. Correlation between DF Case Counts and Pilgrimage Variables

Hajj week timing: There were weak negative correlations between *Hajj week* and DF cases at 1-, 2-, 4-, 5-, and 6-week lags, with the strongest significant correlation at a 4-week lag (r = −0.1) (Figure 4d).

Ramadan timing: There was a positive correlation between *Ramadan* and DF cases at a 1-week lag (r = 0.11) and significant negative correlation from 6 to 12 weeks. The strongest significant negative correlation was at a 12-week lag (r = −0.2) (Figure 4d).

Number of Hajj pilgrims the previous year: There was a weak negative correlation between the number of DF cases and the *number of pilgrims* during the Hajj pilgrimage the previous year (r = −0.11)

Proportion of foreign pilgrims the previous year: No significant correlations.

Table 1 lists the variables included in the final model, including selected bivariate analysis results.

### 3.3. Predictive Models

**Figure 5:**
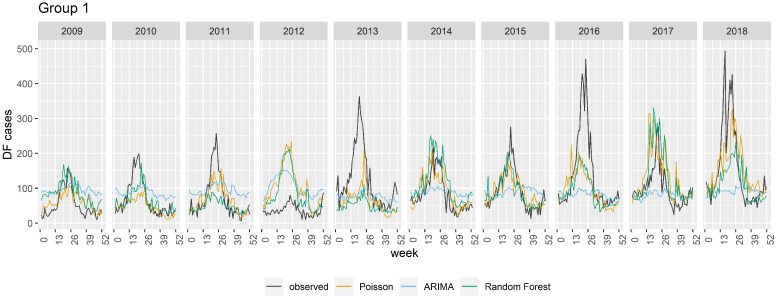

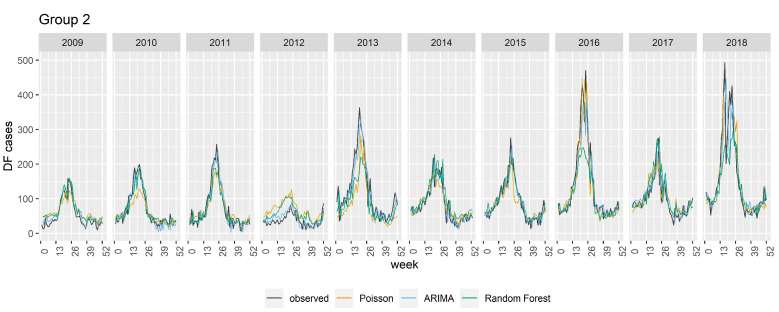
Line graph of observed vs. model predicted DF cases in Jeddah as predicted by Poisson, ARIMA, and random forest regression models. Group number refers to the group of covariates used in the models. Note: for the purpose of visual clarity, the number of cases was capped at 500.

The model performance based on R^2^ and RMSE is averaged for all years and summarized in Table 2. Overall, when Group 1 covariates were used and the *number of cases the previous week* was not included, the RF model performed the best (average R^2^ = 0.62 and RMSE = 43.3) and ARIMA the worst (average R^2^ = 0.26 and RMSE 54.8). When Group 2 covariates were used and the *number of cases the previous week* was included, all models exhibited greater skill, with ARIMA performing the best (average R^2^ = 0.78). The models’ predictive abilities were lower for 2017 and 2018 (R^2^ = 0.56–0.77), and the lowest for 2012 (R^2^ = 0.39–0.55). The R^2^ and RMSE for all models for each year are summarized in Table A1 in Technical Appendix A. Figure 5 illustrates the models’ predictive performance using both covariate groups.

## 4. Discussion

DF ecology in the Arabian Peninsula has not been well described and has two unusual elements: the region’s aridity [4] and the unique large annual religious pilgrimages that bring in people from other endemic regions [7]. This is one of the first model-based investigations of DF epidemiology in Saudi Arabia. The ultimate goal of this work is to develop a predictive model that could facilitate early warning and intervention to reduce future infections. While overall the RF model performed the best, both the ARIMA and Poisson regression models lend insights into the environmental and social factors affecting the epidemic and allows us to examine biologic plausibility and other factors that the black box RF model can obscure. Our findings suggest that our predictive models have sufficient skill to be used in prevention and control efforts.

The seasonal distribution of DF in our dataset has previously been described locally [5,11,23], and globally [9,10,32], and as previously mentioned, largely exhibited the effect of weather on vector life cycle dynamics. The slight dips in the trend at 2–3 years (Figure 3) have previously been discussed in the literature. Jayaraj et al., (2019) explained this phenomenon of ebb and flow in DF epidemiology by the replacement of the dominant circulating viral serotype with another serotype resulting in a process of virus extinction and reinvasion termed “clade replacement” [33], consistent with Saudi Arabia’s experience.

We found a moderately strong association with temperature variables, which is supported by the literature. Temperature acts on multiple components of the ecologic pathway, including viral replication, mosquito oviposition, and larval development and density, with higher temperatures favoring these processes [1,9,34]. Wu et al. (2009) contend that minimum temperature was the most critical for mosquito survival and development [35]. The literature also suggests that average temperatures between 20 and 30 °C are most suitable for *Ae. aegypti* population growth [1,5,9,11]. Morin et al. (2013) emphasize that this association needs to be considered in the context of the local climate. For at least a third of the year, average temperatures in this region are over 30 °C and can reach up to 40 °C [36]. This might explain the shift we see in the relationship between temperature and dengue incidence with increasing lag. At lower temperatures, the relationship between temperature and DF cases is positive; however, as temperatures continue to rise past ~32 °C conditions become detrimental to the mosquito [16,17,18,19,35], inflecting the relationship.

The relationship with humidity variables followed a similar pattern. While collinearity between the two weather variables likely contributed to the association, humidity also plays an independent role, as it is associated with increased mosquito feeding, survival, and egg development [9]. Lab studies have shown that although higher humidity generally favors the mosquito life cycle, higher temperatures and moderate humidity levels (28 °C and 50 to 55% relative humidity (RH) are better suited to the vector compared to environments of very high RH and slightly lower temperature (25 °C and 85 to 90%) [37]. In studies investigating DF in Guangzhou, China, both Wu et al., (2018), and Xiang et al., (2017) found that very high RH has a negative relationship with DF incidence [17,36]. Observed variability in the DF–humidity relationship has been explained in part by climatic differences. For example, in tropical regions like Indonesia where humidity is very high year round (70–80%), no significant association was observed, whereas areas with more moderate humidity reported significant positive associations [17].

Although some studies have reported an association between precipitation and DF [33], it is debatable whether this factor is significant in urban areas where the primary vector breeding habitats may be in indoor containers [32]. The weaker rainfall association we found is likely attributable to rain’s rarity in this region. Water storage behaviors in response to water shortages are more likely to influence mosquito breeding habitats [5,7]. Unfortunately, we do not have access to any water storage data for the region.

The positive correlation between Ramadan and DF is likely due to crowding and increased movement in the Jeddah/Makkah region with the exponential increase in the number of domestic pilgrims during the holy month of Ramadan, the most common pilgrimage time. In 2016, the number of domestic Umrah pilgrims was 16.5 million, nearly half of whom visited during Ramadan [25].

Curiously, like Siddiq et al. [31], we did not find an association between DF and the Hajj timing. There are several potential explanations. First, reporting may decrease during the Hajj, as local health resources are focused on the large influx of visitors. As DF typically presents as mild non-specific symptoms, this may lead to fewer health center visits and thus less reporting during this busy time. Second, active DF cases in Hajj pilgrims may be identified and isolated by health screenings before and upon entering the country, including screening by thermal cameras at Jeddah international airport [38]. Similarly, sick potential pilgrims may self-select, as the Hajj pilgrimage is physically demanding and unlikely to be attempted by someone who is ill. In addition, the virus extinction reinvasion concept, described earlier, could also contribute to the negative correlation between DF cases and the number of pilgrims the previous year. Finally, it is our hypothesis that the negative association with the timing of pilgrimage events is most likely an artifact of the seasonality of these events. The timing of the Hajj in the last 10 years has occurred in early fall, when DF incidence is historically low. Ramadan has also failed to coincide with the peak DF season in the last 10 years. By 2025, the Ramadan and Hajj events will take place between March and June. Notably, DF first emerged in 1993, when these two holy events also took place during the spring. Additional data, including viral serotyping, and further analyses, such as hindcasting to the period of DF emergence in the region, would be required to further evaluate causal mechanisms linking the pilgrimages and DF incidence over the past 25 years.

While overall the RF model had the highest predictive ability, both the ARIMA and Poisson models also contributed to in our analysis by providing clues regarding the various environmental and social factors impacting DF epidemiology in the region. Poisson regression has been standard for studying the impact of weather on DF but has been supplanted by other approaches in recent years. In this study, the Poisson model performed well overall but was not able to capture the magnitude of DF peaks. ARIMA models are also commonly used [32,39] and, while ideal for tackling large datasets, are also known for their sensitivity to outlier data points and poor handling of missing values and multicollinearity [32]. Here, the ARIMA model performed very well with Group 2 covariates but less so when the variable of the *number of cases the previous week* was not included, which is unsurprising given ARIMA’s reliance on historical data. This is an issue when attempting forecasts in places where there are limited or no surveillance data.

The RF model’s overall superior predictive ability, with or without the *number of cases the previous week*, likely derives from the approach’s ability to handle outlier data [32] and better capture non-linear relationships [40]. In assessing DF prediction methods, researchers have emphasized the superiority of tree-based and support vector regression (SVR) machine-learning models compared to those utilizing linear regression [32,41]. In China, Guo et al. found SVR to be the most accurate [41], and Carvajal et al. demonstrated the advantage of an RF approach compared to a variety of other models [32]. A study in Jeddah found that machine-learning methods with environmental and weather variables were adept at predicting DF outbreak locations [31]. Tree-based methods have also been utilized to project the geospatial expansion of the disease vector while subject to varying climate change scenarios. Machine-learning methods are particularly suited to investigate questions where in spite of accumulating large amounts of data many theoretical knowledge gaps persist [40]. Although the RF approach has been shown to be promising in DF prediction, the complex role that several environmental and population factors play on disease incidence leads to differing findings in the relationship between climate and DF in various locations [30].

All of the approaches struggled with some aspect of the relationship between weather and DF, particularly epidemic peaks, likely due to several factors. First, the actual relationships may vary over time. For instance, Xiang et al., (2017) described the relationship between weather and DF as linear up to a specific threshold, beyond which the association is less straightforward and more nuanced [17,37]. Second, some plausible drivers are unobserved, e.g., urban microclimate conditions [40]. Lastly, there may be other overlooked contributing factors at work not included in our model whose effect is more profound during the peak of the epidemic. This is supported by the fact that even the nonlinear RF model struggled to accurately represent the magnitude of the contagion during the seasonal peak.

Our study has several limitations. The first is missing DF count data. We found 19 missing days, from 2 May to 19 May 2018, in the Jeddah dataset (Figure 1). This likely influenced the magnitude of the correlation between the observed DF cases and the cases predicted by either model, but had no bearing on the comparison between the two models. We also suspect significant under-reporting, observed in many countries [10], due to asymptomatic cases, misdiagnosis of mild DF cases, or changes in reporting standards or rates of DF testing over the study period. Additionally, as noted, we have no data on other factors known to affect dengue ecology like water storage, household density, and the prevalence of window screens and air conditioning prevalence that might affect the extent of suitable habitat or transmission dynamics. Lastly, our findings may not be generalizable, as statistical models are usually very location specific [39].

## 5. Conclusions

DF, endemic in the Arabian peninsula, has complex ecology that is strongly affected by local environmental and social factors [7]. Local virus serotypes, immunity patterns, population demographics and movement, and intervention programs affect DF epidemiology [30]. DF ecology in Saudi Arabia was not well characterized prior to our study. We found that temperature, humidity, and, to a much lesser extent, rainfall affect DF incidence there. Additionally, the two main pilgrimages involving the city of Makkah might also play a role in DF incidence, but how and to what extent remains unclear.

We found that a nonlinear machine-learning approach had better prediction accuracy, particularly in the absence of accurate surveillance data. These models could have varying applications depending on the timing of the application. For example, the ability to predict disease incidence two or three months in advance potentially allows for primary prevention interventions, such as vector control, including eliminating mosquito breeding habitats in the form of household water containers. Whereas, predicting the disease a week or two in advance gives medical personnel time to prepare for the influx of patients.

Further investigation is needed to better understand the role various environmental and population factors play in DF incidence in this sparsely studied geographic area and to better prepare the region’s healthcare system to anticipate and intervene to reduce the spread of this disease.

## Figures and Tables

**Table 1:** Strength of the association between independent variables and DF cases based on a bivariate regression analysis.

Variable Type	Variable Name	Lag Period (Weeks)	Pearson Correlation Coefficient (R)	*p*-Value
Weather	*Mean maximum temperature*	8	−0.19 (−0.27, −0.11)	1.36^−5^
	*Mean minimum temperature*	1	0.35 (0.27, 0.42)	<2.2 × 10^−16^
	*Mean relative humidity*	1	−0.51 (−0.57, −0.44)	<2.2 × 10^−16^
	*Number of rainy days*	7	0.09 (0.008, 0.18)	0.033
Pilgrimage	*Hajj week*	4	−0.1 (−0.18, −0.01)	0.024
	*Ramadan*	1	0.11 (0.02, 0.19)	0.016
	*Ramadan*	12	−0.2 (−0.28, −0.11)	5.39 × 10^−6^
	*Number of pilgrims the previous year*	N/A	−0.11 (−0.19, −0.02)	0.014
Other	*Year*	N/A	0.33 (0.26, 0.41)	2.83 × 10^−15^
	*Number of cases the previous week*	N/A	0.91 (0.89, 0.92)	<2.2 × 10^−16^

N/A not applicable.

**Table 2:** Correlation measure (R^2^) and error (RMSE) for each regression model with each of the 2 covariate groupings averaged for all years 2009–2018.

Covariate Group	Regression Model	R^2^	RMSE
1	Poisson	0.51	42.9
	ARIMA	0.26	54.8
	Random Forest	0.62	43.3
2	Poisson	0.74	28.1
	ARIMA	0.79	20.5
	Random Forest	0.78	24.1

**Figure A1:**
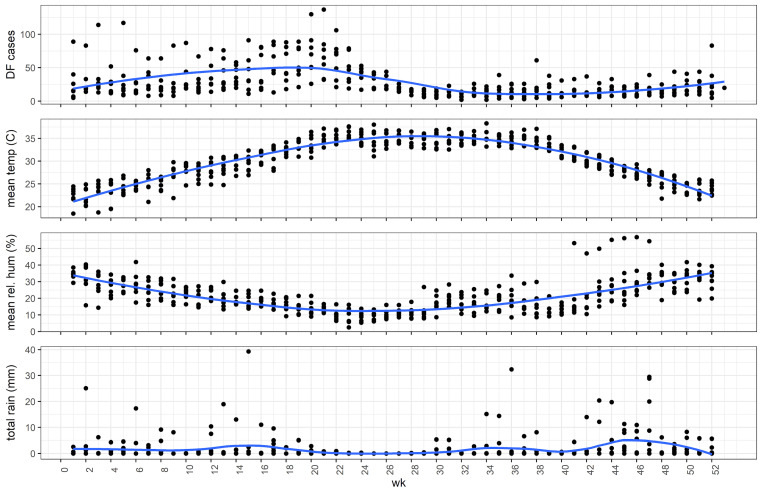
Distribution of DF cases, temperature, humidity, and rainfall throughout the year in Makkah, based on average measurements for 2012 to 2018. The blue line represents the Locally Weighted Scatterplot Smoothing (LOWESS).

**Figure A2:**
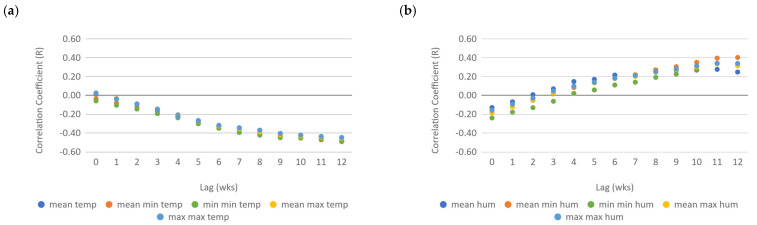

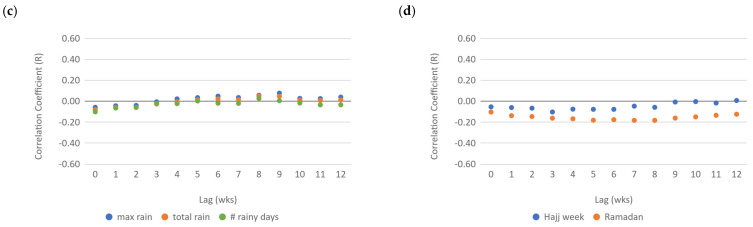
Results of the univariate analysis between DF cases and weather and population variables in the city of Makkah between 2012 and 2018. (**a**) Correlation with temperature variables. (**b**) Correlation with humidity variables. (**c**) Correlation with precipitation variables. (**d**) Correlation with pilgrimage variables.

**Figure A3:**
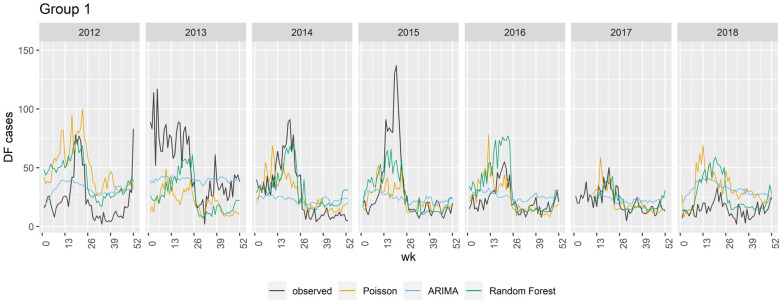

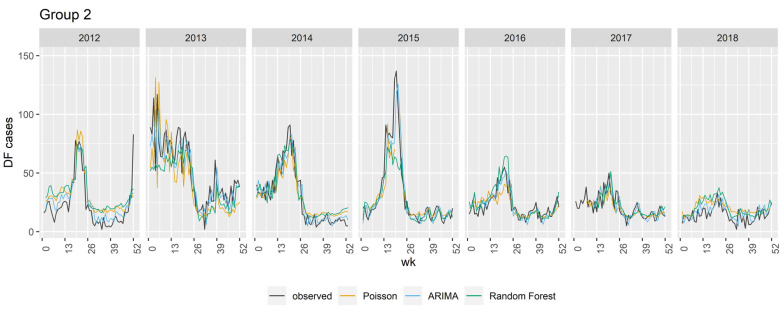
Line graph of observed vs. model predicted DF cases in Makkah as predicted by Poisson, ARIMA, and random forest regression models. Group number refers to the group of covariates used in the models. Note: For the purpose of visual clarity the number of cases was capped at 150.

## Data Availability

The health data used in this study were provided by the Saudi Ministry of Health through a formal request process and cannot be shared without authorization by the Saudi Ministry of Health. The weather GLDAS data are publicly available online through NASA’s Goddard Earth Sciences Data and Information Services Center. Saudi population and pilgrimage data are publicly available online from the Saudi Authority for Statistics website.

## Appendix A

**Table A1:** Correlation measure (R^2^) and error (RMSE) for each regression model and each year (city of Jeddah). Group number refers to the group of covariates used in the models.

Year Validated	Covariate Group	Regression Model	R^2^	RMSE
2009	1	Poisson	0.53	23.6
		ARIMA	0.37	49.5
		Random Forest	0.67	36.1
	2	Poisson	0.82	19.3
		ARIMA	0.86	13
		Random Forest	0.85	16.9
2010	1	Poisson	0.56	30.8
		ARIMA	0.31	45.2
		Random Forest	0.61	25.5
	2	Poisson	0.85	20.4
		ARIMA	0.87	15.3
		Random Forest	0.85	15.2
2011	1	Poisson	0.62	28.1
		ARIMA	0.48	50.8
		Random Forest	0.68	33
	2	Poisson	0.89	20.4
		ARIMA	0.85	19.2
		Random Forest	0.88	17.6
2012	1	Poisson	0.33	67.2
		ARIMA	0.23	70.1
		Random Forest	0.33	52.5
	2	Poisson	0.39	31.6
		ARIMA	0.55	11.6
		Random Forest	0.4	22.2
2013	1	Poisson	0.45	63.2
		ARIMA	0.26	61.6
		Random Forest	0.59	75.5
	2	Poisson	0.77	46.5
		ARIMA	0.85	25.8
		Random Forest	0.78	38.7
2014	1	Poisson	0.57	27.9
		ARIMA	0.51	38.3
		Random Forest	0.74	34.9
	2	Poisson	0.81	17.5
		ARIMA	0.8	18.2
		Random Forest	0.77	20.9
2015	1	Poisson	0.64	26.3
		ARIMA	0.005	46.1
		Random Forest	0.75	18.6
	2	Poisson	0.8	20.1
		ARIMA	0.82	18.1
		Random Forest	0.89	14
2016	1	Poisson	0.48	60.2
		ARIMA	0.23	72.3
		Random Forest	0.77	54.1
	2	Poisson	0.83	29.8
		ARIMA	0.86	27.1
		Random Forest	0.87	36.9
2017	1	Poisson	0.41	45.9
		ARIMA	0.06	37.8
		Random Forest	0.6	42.4
	2	Poisson	0.67	23.2
		ARIMA	0.7	21.7
		Random Forest	0.7	21.5
2018	1	Poisson	0.51	56.2
		ARIMA	0.07	75.9
		Random Forest	0.45	60.1
	2	Poisson	0.56	52.3
		ARIMA	0.71	35
		Random Forest	0.77	36.7

## Appendix B

**Table A2:** Strength of the association between variables in Covariate Groups 1 and 2 with DF cases in Makkah based on a bivariate regression analysis.

Variable Type	Variable Name	Lag Period (Weeks)	Pearson Correlation Coefficient (R)	*p*-Value
Weather	*Mean maximum temperature*	8	−0.4	2.09^−15^
	*Minimum minimum relative humidity*	1	−0.17	0.001
	*Mean minimum relative humidity*	8	0.28	<8.92 × 10^−8^
Pilgrimage	*Ramadan*	8	0.11	0.016
	*Proportion of foreign pilgrims the previous year*	N/A	−0.4	9.33 × 10^−16^
	*Number of pilgrims the previous year*	N/A	0.25	8.01 × 10^−7^
Other	*Year*	N/A	−0.28	3.44 × 10^−8^
	*Number of cases the previous week*	N/A	0.87	<2.2 × 10^−16^

N/A not applicable.

**Table A3:** Correlation measure (R^2^) and error (RMSE) for each regression model averaged for all years 2012–2018 (Makkah). Group number refers to the group of covariates used in the models.

Covariate Group	Regression Model	R^2^	RMSE
1	Poisson	0.34	14
	ARIMA	0.21	16.3
	Random Forest	0.5	14.3
2	Poisson	0.57	8.9
	ARIMA	0.61	7.7
	Random Forest	0.63	9.4

**Table A4:** Correlation measure (R^2^) and error (RMSE) for each regression model in the two covariate groupings for each year (Makkah).

Year Validated	Covariate Group	Regression Model	R^2^	RMSE
2012	1	Poisson	0.43	16.2
		ARIMA	0.38	16.3
		Random Forest	0.55	20.5
	2	Poisson	0.64	8.7
		ARIMA	0.69	6.7
		Random Forest	0.67	12.7
2013	1	Poisson	0.21	23
		ARIMA	0.03	23.9
		Random Forest	0.42	26
	2	Poisson	0.44	18
		ARIMA	0.56	13.4
		Random Forest	0.64	15.8
2014	1	Poisson	0.57	13.7
		ARIMA	0.39	18.1
		Random Forest	0.6	13.7
	2	Poisson	0.84	9.7
		ARIMA	0.85	7.2
		Random Forest	0.84	9.1
2015	1	Poisson	0.31	18.2
		ARIMA	0.13	22.2
		Random Forest	0.53	14.9
	2	Poisson	0.78	10
		ARIMA	0.85	7.7
		Random Forest	0.77	10.6
2016	1	Poisson	0.25	9.5
		ARIMA	0.09	8.8
		Random Forest	0.59	9.4
	2	Poisson	0.55	6
		ARIMA	0.63	5.7
		Random Forest	0.68	5.5
2017	1	Poisson	0.42	8.3
		ARIMA	0.36	8.5
		Random Forest	0.5	5.4
	2	Poisson	0.48	5.6
		ARIMA	0.46	6.2
		Random Forest	0.47	5.6
2018	1	Poisson	0.16	8.9
		ARIMA	0.1	16
		Random Forest	0.29	10
	2	Poisson	0.25	4.6
		ARIMA	0.26	6.8
		Random Forest	0.32	6.8
